# Cytoprotective Effects of Cell-Permeable Bifunctional Antioxidant Enzyme, GST-TAT-SOD, against Cisplatin-Induced Cell Damage

**DOI:** 10.1155/2017/9530791

**Published:** 2017-11-30

**Authors:** Jianru Pan, Lingling Li, Lili Liang, Huocong He, Ying Su, Xiangling Wang, Shutao Liu

**Affiliations:** ^1^College of Biological Science and Engineering, Fuzhou University, No. 2 Xue Yuan Road, University Town, Fuzhou, Fujian 350108, China; ^2^Euler Genomics (Beijing) Co., Ltd., No.8 Life Science Park Road, Zhongguancun Life Science Park, Beijing 102206, China; ^3^Laboratory of Radiation Oncology and Radiobiology, Fujian Cancer Hospital & Fujian Medical University Cancer Hospital, Fuzhou, Fujian 350014, China; ^4^Fujian Key Laboratory of Tumor Translational Cancer Medicine, Fuzhou, Fujian 350014, China

## Abstract

GST-TAT-SOD, a cell-permeable bifunctional antioxidant enzyme, is a potential selective radioprotector. This study aimed to investigate the cytoprotective activity of GST-TAT-SOD against cisplatin-induced damage. The current study showed that cisplatin induced the formation of reactive oxygen species in normal L-02 cells. GST-TAT-SOD (2000 U/mL) executed its antioxidant role by directly scavenging excess intracellular free radicals and augmenting cellular antioxidant defense such as reducing MDA level, enhancing the SOD activity, GST activity, and T-AOC. Thus, it suppressed the growth inhibition and apoptosis of cisplatin-treated normal cells. Meanwhile, the growth inhibition of tumor cells (SMMC-7721) caused by cisplatin was unaffected by GST-TAT-SOD pretreatment. GST-SOD, as a comparison, seemed to be powerless for related indicators as it could not enter into cells without cell-permeating peptide. These results suggest that GST-TAT-SOD might be a potential cytoprotective agent for cisplatin-induced side effects.

## 1. Introduction

Cisplatin, *cis*-diamminedichloroplatinum (II) or CDDP, is the most potent chemotherapy drugs widely used against the spread of solid tumors including tumors of the ovary, cervix, bladder, lung, head, and neck in adults [1, 2]. Clinical success of cisplatin and its derivatives determines considerable effort to develop other effective metal-based anticancer compounds [3–5]. However, its side effects in normal tissues limit its clinical application. These side effects which are also observed at therapeutic doses include gastrointestinal effects (e.g., nausea and vomiting), bone marrow suppression, ototoxicity, neurotoxicity (e.g., peripheral neuropathy), hepatotoxicity, and genotoxicity [6].

The mechanism of action of cisplatin is mainly based on DNA damage. It enters into cells, binds to DNA, and, consequently, induces apoptosis and inhibits cell growth [7]. Most metals can generate reactive oxygen species (ROS). Except for DNA damage, many data suggest that cisplatin also induces ROS that trigger cell death [8–11]. The ROS levels depend on the concentration of cisplatin and the duration of exposure [12]. It is reported that cisplatin-induced ROS is responsible for the severe side effects of cisplatin therapy such as hepatotoxicity [12].

In this direction, as safeguards against the accumulation of ROS, antioxidant enzymes would be a kind of promising protector against cisplatin-induced injury. Superoxide dismutases (SODs) catalyze the dismutation of superoxide into oxygen (O_2_) or hydrogen peroxide (H_2_O_2_) and serve key antioxidant roles as superoxide is one of the main reactive oxygen species in the cells. However, they are hard to enter into the cells due to their large molecular weights. Protein transduction domain (PTD) TAT (YGRKKRRQRRR) derived from HIV-1 Tat protein can carry larger molecules such as oligonucleotides and full-length proteins across cellular membranes and has been proven useful in delivering biologically active cargoes both in vitro and in vivo models [13].

Therefore, in our previous works, we constructed cell-permeable monofunctional antioxidant enzyme SOD-TAT with the fusion of human CuZn-SOD and TAT peptide [14]. We reported that SOD-TAT has a significant protective effect on radiation side effect induced by extra ROS both in vitro and in vivo [15, 16]. To eliminate more types of free-radical species induced by irradiation, glutathione S-transferase (GST) was further inserted into the fusion protein and formed a bifunctional antioxidant enzyme GST-TAT-SOD. GSTs catalyze the conjugation of the reduced form of glutathione (GSH) to xenobiotic substrates for detoxification. As expected, GST-TAT-SOD has a better radioprotective effect than SOD-TAT [16]. The former represents remarkable protective effects on irradiated normal liver cells and minimal effect on irradiated hepatoma cells [16]. Moreover, we recently reported that GST-TAT-SOD has an overall protective effect on the whole body-irradiated mice [17].

The current study investigated the cytoprotective effects of GST-TAT-SOD against cisplatin-induced cell damage.

## 2. Materials and Methods

### 2.1. Materials

*E.coli* strains with the recombinant plasmid of GST-TAT-SOD and GST-SOD were obtained from the Institute of Biotechnology, Fuzhou University (Fujian, China). Malondialdehyde (MDA), SOD, total antioxidant capacity (T-AOC), and glutathione S-transferase (GST) reagent kits were purchased from Nanjing Jiancheng Bioengineering Co. Ltd. (Jiangsu, China). Micro BCA™ Protein Assay Kit was purchased from Thermo Scientific (USA). RPMI 1640 and fetal bovine serum were purchased from HyClone and Gibco (USA), respectively. Methyl thiazolyl tetrazolium (MTT) and 2′, 7′-dichlorodihydrofluorescein diacetate (DCFH-DA) were purchased from Sigma (USA). FITC Annexin V apoptosis detection kit was purchased from BD Pharmingen (USA). Cisplatin was purchased from Haosen Pharmaceutical Group Co., Ltd. (Jiangsu, China). All other chemicals were of analytical purity.

### 2.2. Cell Cultures

Human normal liver cell line L-02 and hepatoma cell line SMMC-7721 are available from Shanghai Institute of Biochemistry and Cell Biology (SIBCB). Cells were cultured in RPMI 1640 (HyClone), supplemented with 10% fetal bovine serum (Gibco), 100 U/ml penicillin and 100 mg/ml streptomycin (Gibco) at 37°C in a 5% CO_2_ humidified chamber.

### 2.3. Preparation of GST-TAT-SOD

GST-TAT-SOD and GST-SOD were prepared according to the method of our previous work [16]. The concentration, SOD activity, and GST activity of the purified proteins were determined by BCA protein assay kit (Thermo, USA), SOD, and GST reagent kits (Jiangsu, China), respectively. The SOD activity and GST activity of purified GST-TAT-SOD were 2476 U/mL and 766 U/mL, respectively. Purified proteins were concentrated and dialyzed for subsequent experiments.

### 2.4. Cytotoxicity Assay

MTT, 3-[4,5-dimethylthiazol-2-yl]-2,5-diphenyl tetrazolium bromide, is reduced by active mitochondrial dehydrogenases of living cells to formazan. The conversion can be measured at 570 nm spectrophotometrically. Nearly 2 × 10^5^ L-02 cells and SMMC-7721 cells were seeded per well in 96-well plates. Then, cells were treated with various concentrations of GST-TAT-SOD or GST-SOD. After incubation for 3 h, 0.7 *μ*g/mL cisplatin was added to each well and incubated for 24 h (three replicates each). Then, cell viability was calculated by a colorimetric assay using MTT (Sigma, USA).

### 2.5. Biochemical Estimations

L-02 cells in logarithmic growth phase were trypsinized and counted with a hemocytometer. Approximately 1.75 × 10^5^ cells were seeded into 75 cm^2^ flasks. The experiments were performed in triplicate, and tests were carried out on the following different cell groups. The first group (CON) was untreated and the second group (CDDP) was treated with 0.7 *μ*g/mL cisplatin alone. GTS + CDDP group and GS + CDDP group were pretreated with indicated concentrations of GST-TAT-SOD or GST-SOD, respectively, for 3 h before injured by cisplatin.

After adding cisplatin, cells were further cultured for 24 h at 37°C. Then, cells were lysed using PBS containing 0.5% Triton X-100. The cell lysates were removed and centrifuged at 13,000 rpm for 15 min at 4°C. The SOD activity, GST activity, T-AOC, and MDA level in the supernatants were determined using their corresponding diagnostic reagent kits (Nanjing Jiancheng Bioengineering) spectrophotometrically according to the manufacturer's instructions. The protein contents of the lysates were determined by using BCA protein assay kit (Thermo, USA).

### 2.6. ROS Assay

The conversion of DCHF-DA (Sigma, USA) was used to assess the production of endogenous total ROS [18]. Cells (approximately 1.75 × 10^5^ cells per well) were plated in triplicate, grouped as described in section 2.5, pretreated with or without proteins, and treated with 0.7 *μ*g/mL cisplatin for 24 h. After incubation, cells were rinsed with PBS, added 10 *μ*M DCFH-DA, and incubated for 0.5 h at 37°C.

Then, cells from the different groups were lysed as described in Section 2.5 and the fluorescence in the supernatants were read with a Synergy H4 Hybrid Multi-Mode Microplate Reader (BioTek Instruments, Winooski, VT, USA). The protein contents of the cell lysates were determined by BCA protein assay kit (Thermo, USA) following the manufacturer's directions. Fluorescence was corrected for background signal and normalized for protein content and expressed as fluorescence/mg protein.

### 2.7. Apoptosis Assays

Approximately 7 × 10^5^ cells were seeded into 75 cm^2^ flasks and grouped as described in Section 2.5. Then, cells were pretreated with or without proteins and incubated for 24 h with 0.7 *μ*g/mL cisplatin. Apoptotic cells were identified by the Annexin V-FITC Apoptosis Detection Kit (BD Pharmingen, San Diego, CA, USA) according to the manufacturer's instructions using a FACScan flow cytometer.

### 2.8. Statistical Analysis

Statistical analysis of all data was performed using Excel. The results are reported as means ± S.E. or S.E.M. The *P* values were determined using the Student two-tailed *t*-test, and *P* < 0.05 or *P* < 0.01 was considered statistically significant.

## 3. Results

### 3.1. Biological Effect of GST-TAT-SOD on the Cell Viability of Cisplatin-Treated Cells

**Figure 1:**
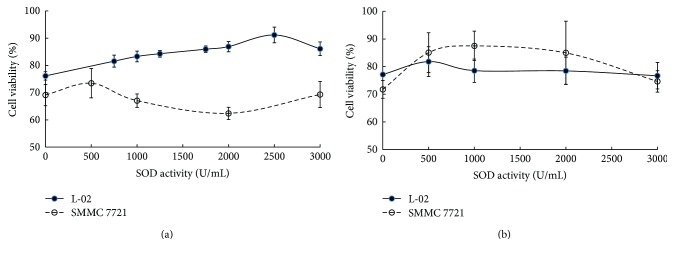
Biological effect of fusion proteins on the cell viability of cisplatin-treated cells. Nearly 2 × 10^5^ cells were seeded per well in 96-well plates and treated with various concentrations of GST-TAT-SOD (a) or GST-SOD (b). After incubation for 3 h, 0.7 *μ*g/mL cisplatin was added to each well and incubated for 24 h (three replicates each). The bars indicate the means ± SD (*n* = 3).

It can be seen from Figure 1 that GST-TAT-SOD shows an entirely different biological effect on normal cells or cancer cells injured by cisplatin. It had a protective role in L-02 cells against the cytotoxicity induced by cisplatin (Figure 1(a)). We found that the percentage of viable cells increased continuously among L-02 cells at lower doses of GST-TAT-SOD, the maximal vitality increased by about 15% at 2500 U/mL, and a progressive decrease in cell viability at higher protein concentrations. By contrast, GST-TAT-SOD had little effect on the cell viability of cisplatin-treated SMMC-7721 cells (Figure 1(a)). They presented minimal viability decreased by about 7% at 2000 U/mL GST-TAT-SOD pretreatment. To sum up, 2000 U/mL GST-TAT-SOD pretreatment could reduce the growth inhibition of L-02 cells induced by cisplatin while that of SMMC-7721 cells were enhanced in the meantime. Hence, 2000 U/mL GST-TAT-SOD pretreatment was used in the experiments. Compared with GST-TAT-SOD, GST-SOD seemed to provide better protection for cisplatin-treated SMMC-7721 cells than L-02 cells (Figure 1(b)).

### 3.2. Biochemical Estimations

**Figure 2:**
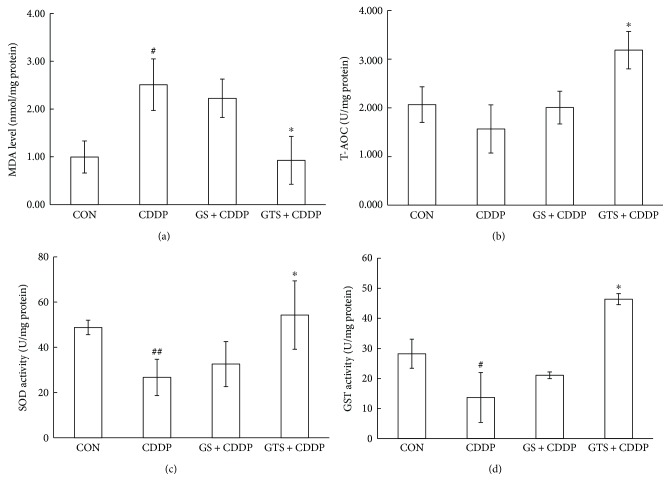
Biochemical estimations of L-02 cells treated with cisplatin. (a) MDA level, (b) T-AOC, (c) SOD activity, and (d) GST activity. Cells were treated with protein (2000 U/mL) for 3 h followed by 0.7 *μ*g/mL cisplatin. After treatment, the cells were further cultured for 24 h at 37°C. Then, the cells were lysed and centrifuged, and the antioxidant activities and protein contents in the supernatants were determined subsequently. The bars indicate the means ± SD (*n* = 3). The CDDP group was compared with the control group (^#^*P* < 0.05, ^##^*P* < 0.01). The pretreated group was compared with the CDDP group (^∗^*P* < 0.05).

As is shown in Figure 2, reductions in the activity of SOD (*P* < 0.01), GST (*P* < 0.05), and T-AOC (*P* > 0.05) and a significant increase in the level of MDA (*P* < 0.05) were observed in the CDDP group 24 h after cisplatin treatment. GST-TAT-SOD pretreatment remarkably decreased the level of MDA by about 63% (*P* < 0.05), enhanced the SOD activity by about 103% (*P* < 0.05), GST activity by about 239% (*P* < 0.05), and T-AOC by about 103% (*P* < 0.05) of cisplatin-damaged cells. GST-SOD pretreatment seemed to be slightly protective on these indicators, but there was no significance between the CDDP group and GS + CDDP group.

### 3.3. ROS Assay

**Figure 3:**
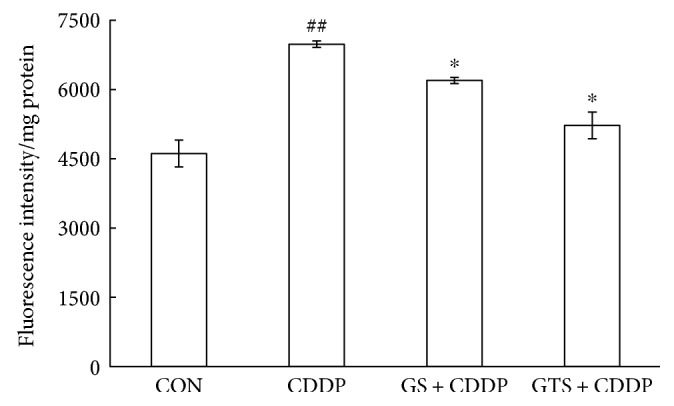
DCFH-DA detection of total ROS in L-02 cells treated with cisplatin. Cells were plated in triplicate, pretreated with or without proteins, and treated with 0.7 *μ*g/mL cisplatin for 24 h. After incubation, cells were washed with PBS and incubated with 10 *μ*M DCFH-DA for 0.5 h at 37°C. Fluorescence was corrected for background signal and normalized for protein content and expressed as fluorescence/mg of protein. The bars indicate the means ± SD (*n* = 3). The CDDP group was compared with the control group (^##^*P* < 0.01). The pretreated group was compared with the CDDP group (^∗^*P* < 0.05).

Total ROS in L-02 cells induced by cisplatin was evaluated using DCFH-DA. A significant enhancement by about 51% in ROS was observed in the cells after cisplatin treatment as is shown in Figure 3 (*P* < 0.01). GST-SOD pretreatment reduced the extra ROS by about 11% (*P* < 0.05) while that of GST-TAT-SOD pretreatment reduced by about 25% (*P* < 0.05).

### 3.4. Apoptotic Index

**Figure 4:**
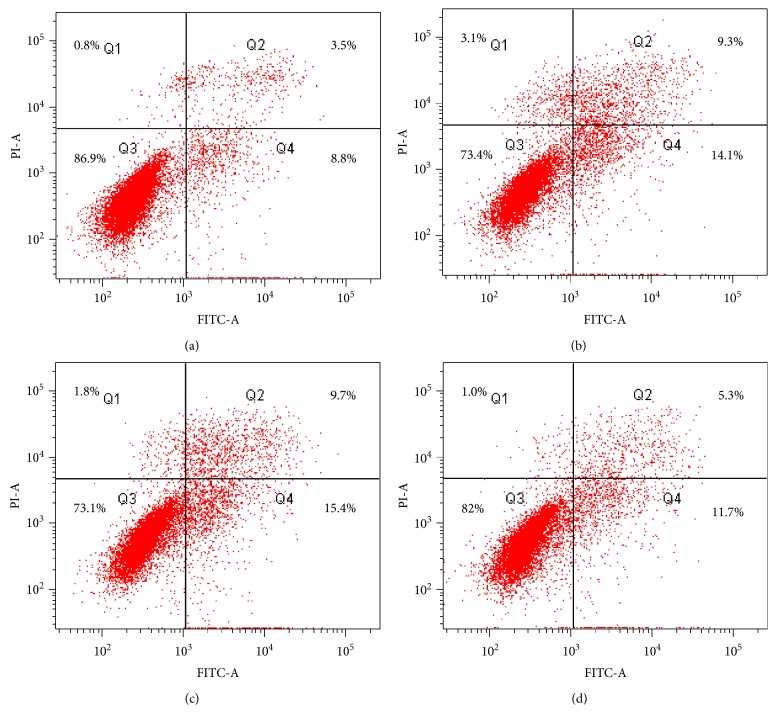
Annexin V/PI analysis of apoptosis in L-02 cells at 24 h after treatment with 0.7 *μ*g/mL cisplatin. Cells were stained with Annexin V FITC and PI and detected by flow cytometry. The lower right quadrant (Annexin V+ PI−) represents early apoptosis, whereas the upper right quadrant (Annexin V+ PI+) represents late apoptosis. (a) CON, (b) CDDP, (c) GS + CDDP, and (d) GTS + CDDP.

To explore whether GST-TAT-SOD could protect L-02 cells against cisplatin-induced apoptosis, Annexin V/PI staining was performed using flow cytometric analysis. As is shown in Figure 4, compared with the CON group, the total apoptotic rate of the CDDP group increased by about 90%. GST-TAT-SOD pretreatment lowered the total apoptotic rate in cisplatin-injured cells by 27%, while GST-SOD pretreatment seemed to be invalid.

## 4. Discussion

Cisplatin is a widely used chemotherapy agent against many solid tumors. However, the dose-independent side effects including ototoxicity, neurotoxicity, and hepatotoxicity placed a health and economic burden on patients [6]. Although the exact mechanism of cisplatin toxicity is not fully understood, multiple studies have shown that the failure of cisplatin-induced DNA damage repair ultimately results in cell death. Besides, the formation of cisplatin-induced ROS and oxidative stress also lead to apoptosis [8–11]. It is reported that antioxidants such as vitamin E, carotenoids, GSH, phytoestrogens, phenols, and polyphenols can prevent the cisplatin-induced mitochondrial production of ROS [19–21]. In that case, enzymatic antioxidants like SOD, catalase (CAT), glutathione peroxidase (GPx), and GST would be more efficient.

We constructed a cell-permeable bifunctional antioxidant enzyme GST-TAT-SOD in our previous works. GST-TAT-SOD retains more than 90% of the enzyme activity under physiological conditions [22]. It can enter into cells by the transduction with TAT-PTD in an active form, scavenge excess production of ROS, balance intracellular oxidant-antioxidant status, and protect the cells against irradiation damage [16]. Besides that, GST-TAT-SOD can effectively transduce into tissues such as the spleen and liver without affecting tissular antioxidant indices and protect normal tissues from radiation damage [16, 17].

Since ROS is one of the causes of cisplatin damage, we wondered whether GST-TAT-SOD could provide a protective effect against cisplatin damage too. In this study, DCFH-DA was used to evaluate intracellular ROS level. Although DCFH-DA was found to be unsuitable for direct detection of specific ROS [23], it can be used as a reference for a global amount of ROS combined with other relevant indicators such as MDA level. MDA level is an indicator of lipid peroxidation and commonly known as a marker of oxidative stress and the increment of free radicals [24]. A remarkable increase of DCF fluorescence and MDA level was observed in the cisplatin-injured L-02 cells, indicating the formation of extra ROS as described in other literature [8–11] (Figures 2(a) and 3). GST-TAT-SOD significantly reduced the elevation of ROS in cisplatin-injured L-02 cells (Figures 2(a) and 3). The SOD activity and GST activity of GST-TAT-SOD pretreated cells both were remarkably increased compared with the cells of the CDDP group. Except for the enhancement of two enzymatic antioxidants, the T-AOC of GST-TAT-SOD pretreated cells was also remarkably elevated. As an important integrative index, T-AOC reflects the total antioxidant capacity in the cells including both enzymatic and nonenzymatic antioxidants. It indicated that bifunctional GST-TAT-SOD could provide comprehensive protection of the endogenous antioxidant system. Therefore, subsequent repression of the growth inhibition and apoptosis in GST-TAT-SOD pretreated cells was not in doubt as the excessive production of intracellular ROS and oxidative stress could lead to apoptosis [8–11] (Figures 1 and 4). GST-SOD, by contrast, gave a much weaker protection effect on all indicators as it could not enter into cells without cell-permeating peptide (Figures 1–4).

The family of GSTs is part of a cellular Phase II detoxification enzymes. Their catalytic functions are conjugating GSH with a diverse range of electrophilic substrates [25]. Thus, they are described for their roles in detoxification and linkage of their expression levels with high cancer drug resistance is thought to reflect enhanced capabilities in breaking down drugs to noncytotoxic metabolites, although no entire cogent explanation for these correlations exists [26, 27]. So, whether the ability of GST-TAT-SOD to significantly enhance GST activity in cisplatin-injured normal cells would lead to tumor resistance to cisplatin?

Our previous works indicated that GST-TAT-SOD is a potent radioprotector for normal cells while making no significant effects on irradiated tumor cells meanwhile [16]. The selectively protected effect of GST-TAT-SOD was observed in the present study again. GST-TAT-SOD suppressed the growth inhibition of normal cells induced by cisplatin at the dose of 2000 U/mL and simultaneously enhanced that of tumor cells (Figure 1).

All of the above suggest that bifunctional GST-TAT-SOD can significantly reduce the chemotherapy- or radiation-induced side effects without impairing the antitumor efficacy of the two therapies. Further studies are needed to confirm the more exact protective mechanism of GST-TAT-SOD for a comprehensive assessment of its potential as an adjunctive therapy with various cancer treatments.

## 5. Conclusions

In summary, this study has demonstrated that GST-TAT-SOD protects normal cells from cisplatin-induced damages as it can decrease extra ROS, maintain the cellular antioxidant system, and suppress the growth inhibition and apoptosis. Furthermore, it does not alter cisplatin-induced cytotoxicity to cancer cells. This dual activity is necessary for a drug to be considered an effective adjunctive therapy for various cancer treatments.
